# Supplementary figures and images for: Effects of stigmata maydis on the methicillin resistant Staphylococus aureus biofilm formation
Source: PeerJ. 2019 Feb 26;7:e6461. doi: 10.7717/peerj.6461 (PMC6396744; doi:10.7717/peerj.6461)

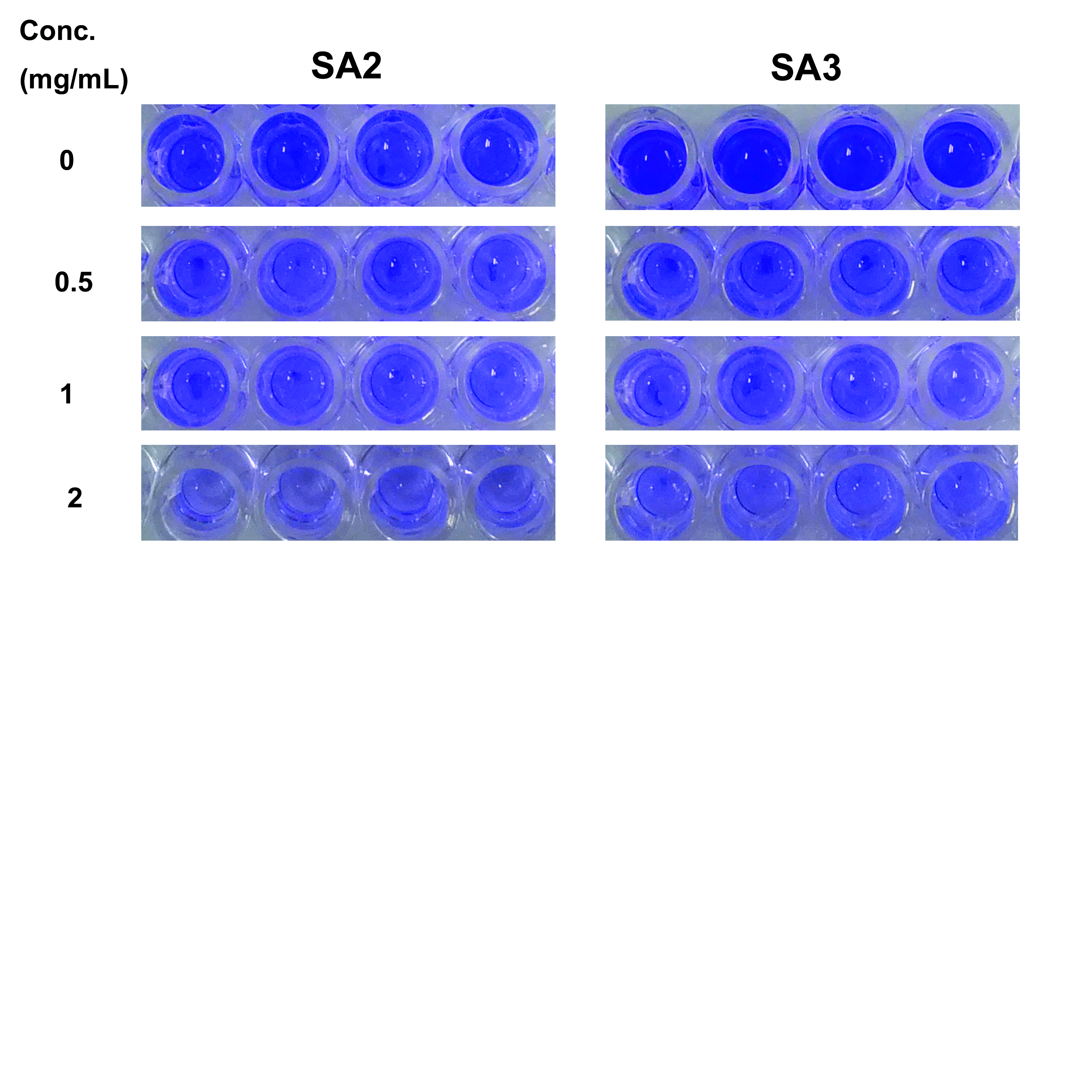

Supplement: Supplemental Information 1 [file peerj-07-6461-s001.zip › Supplemental Files/File 2/IMG_20150808_090624.jpg]
